# Music Performance Improvement Support System Using a Semi-Automated Instrument-Playing Robot with Real-Time Acoustic Analysis and Habit Visualization

**DOI:** 10.3390/s26031053

**Published:** 2026-02-05

**Authors:** Kouki Tomiyoshi, Hiroaki Sonoda, Hikari Kuriyama, Gou Koutaki

**Affiliations:** 1 Graduate School of Science and Technology, Kumamoto University, Kumamoto 860-8555, Japan; tomiyoshi@navi.cs.kumamoto-u.ac.jp (K.T.); sonoda@navi.cs.kumamoto-u.ac.jp (H.S.); kuriyama@navi.cs.kumamoto-u.ac.jp (H.K.); 2 Department of Computer Science and Electrical Engineering, Kumamoto University, Kumamoto 860-8555, Japan

**Keywords:** music performance analysis, player’s habit analysis, woodwind instruments

## Abstract

This paper proposes an acoustic analysis system to help improve saxophone performance skills. The system combines direct support for performance movements by a robot with indirect support by presenting performance information. By sensing the performance audio and performing real-time acoustic analysis, the system presents the learner with information about their performance and their playing habits. The performance information presented to the learner includes pitch, volume, and playing timing. For performance habit analysis, a Markov model with pitch as the state and an internal probability parameter that indicates the quality of the performance evaluation as the pitch transitions are defined. In the experiment, we conducted a pilot study targeting experienced saxophone players and a beginner saxophone player to verify the effectiveness of the proposed system. The experiment showed that the MAE of the played pitch was significantly reduced by using the proposed system.

## 1. Introduction

To play a musical instrument, it is essential to acquire performance actions specific to that instrument. For example, most wind instruments require two primary performance actions: fingering and blowing. Fingering is used to adjust the pitch by changing the effective tube length, whereas blowing involves controlling the embouchure, airflow, and intraoral pressure to regulate pitch, loudness, and timbre. These two performance actions must be internalized through repetitive practice; in most cases, they are not skills that novices naturally possess. Consequently, it is difficult for an inexperienced individual to play such instruments, and a certain period of training is required to acquire the necessary performance actions.

Several approaches exist for training aimed at acquiring performance actions. Among them, the most effective approach is training under the guidance of an instructor with specialized expertise. However, it is often difficult for learners to receive continuous and intensive supervision from an instructor, and, in practice, much of the training is carried out as individual practice. Nevertheless, it is particularly challenging for beginners to accurately assess whether their individual practice is being performed correctly.

A wide range of attempts have been made to enhance the quality of individual practice and thereby support learners in improving their instrumental performance skills [1,2,3,4,5,6,7,8,9,10]. These approaches can be broadly categorized into two types: direct assistance and indirect assistance.

**Figure 1:**
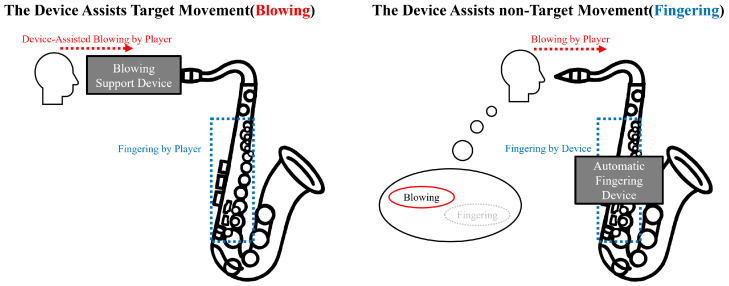
Classification of direct assistance devices. Devices are categorized according to whether they support the target performance actions (**left**) or automate non-target actions to allow learners to focus on other actions (**right**).

Direct assistance devices provide physical support for the learner’s performance actions. From the perspective of assistance strategies, direct assistance devices can be broadly classified into the following two categories. Figure 1 illustrates this classification using examples related to supporting saxophone-blowing actions.

1. Devices that support the performance actions carried out by learners [1,2]. In this category, the supported performance action is the primary target of learning;
2. Devices that automate some of the required performance actions [3,4], enabling learners to practice only the actions that remain.

Indirect assistance devices support skill acquisition by presenting information related to the learner’s instrumental performance actions or the acoustic features of the sound of the performance. Examples of feedback information related to performance actions include hand and finger movements for fingering and airflow or air-velocity information for blowing [5]. Feedback related to acoustic features generally includes pitch, loudness, rhythm, timing, and timbre. Most devices present such feedback through visual or auditory modalities [6,7,8], although some devices provide haptic feedback [9]. A widely used example of an indirect assistance device is the standard electronic tuner, which visually feeds back pitch information to the performer.

Many previous studies have supported instrumental learners through either direct or indirect assistance. However, for performance support aimed at a wide range of learners—from beginners to experienced players—we consider reliance on only one of these approaches to be insufficient. Moreover, most existing indirect assistance devices acquire acoustic features of the performance sound and provide feedback in the form of evaluation scores or feature trajectories. We argue that it is worthwhile to investigate the utility of computing a learner’s performance habits based on such acoustic features and feeding these habits back as part of the learning process.

In this paper, we propose a system that supports the improvement of saxophone learners’ performance skills by integrating both direct and indirect assistance. As an indirect-assistance component, we designed and implemented software that performs real-time analysis of the acoustic features of the performance sound and provides visual feedback. As a direct-assistance component, we employ the semi-automatic saxophone-playing robot developed by Koutaki et al. [4]. Furthermore, to address the previously noted difficulty faced by beginners in accurately evaluating their own playing during individual practice, the proposed system incorporates a function that stores learners’ performance data, extracts characteristic patterns of performance errors, and feeds them back as the “performer’s habits”.

This chapter is organized as follows. Section 1.1 presents the fundamental concept of this study. Section 1.2 describes the technical components we developed. Section 1.3 reviews research related to the proposed system. Section 1.4 discusses the position of this work within the broader research context. Section 1.5 explains the expected effects of using the proposed system.

### 1.1. Basic Idea

A semi-automatic musical instrument performance robot is a device designed to provide any user with an accessible and active performance experience by automating only a subset of the required performance actions. In this study, we focus on how the use of such a semi-automatic performance robot allows learners to concentrate on the performance actions that are not automated by the robot.

In this paper, we target the alto saxophone as the instrument of interest. The semi-automatic saxophone-playing robot automatically performs the fingering actions, allowing learners to concentrate solely on blowing actions. Building on this capability, we propose a system that further enhances the efficiency of training that utilizes the semi-automatic saxophone-playing robot.

To enable learners to engage comfortably in individual practice, the proposed system incorporates gamification into the graphical user interface used for feedback. Specifically, we design a system in which users execute the required performance actions in synchrony with incoming pitch bars, similar to a rhythm game. This allows both the robot-assisted performance of musical pieces and the real-time analysis of the learner’s performance to be achieved simultaneously.

### 1.2. Technical Overview

To realize the above concept, this study develops the following technical components.

#### 1.2.1. Multi-Factor and Real-Time Music Performance Analysis

In this study, we examine a method for providing performers with real-time feedback on the acoustic features of their performance sound. As the acoustic features to be fed back, we adopt pitch, loudness, and note-onset timing, which are considered particularly important for performers. For the extraction of each feature, we selected lightweight algorithms suitable for real-time processing.

#### 1.2.2. Performance Habits Analysis Using Real-Time and Past Performance Results

This study also investigates a method for analyzing a learner’s performance habits. We assume two types of habits in instrumental performance: difficulty in producing specific pitches and difficulty in transitioning from one pitch to another. Based on performance evaluation data and ground-truth data, we construct a Markov model in which each state corresponds to a pitch. For each state transition (i.e., pitch change), we define the “error occurrence rate” and provide this as feedback to the performer.

#### 1.2.3. A GUI That Provides Feedback on Performance Information While Synchronizing with the Robots

Few existing performance-support systems coordinate direct and indirect assistance (details are provided in Section 1.3). In this study, we implement a GUI that provides real-time feedback of the above performance-analysis results while simultaneously controlling the semi-automatic musical-instrument robot. The robot is controlled via MIDI messages exchanged between a PC and the robot’s microcontroller over a wired USB connection. The analysis results are presented visually in an intuitive format so that even beginners can easily understand them.

### 1.3. Related Works

To date, numerous studies have attempted to support musical instrument learners—including beginners—through technological means. However, the target users, instruments, and assistance approaches differ from those of the system proposed in this paper. This section reviews instrument-learning support devices that are technically related to our study.

#### 1.3.1. Direct-Assistance Devices

We first describe examples of direct-assistance devices, which physically support the learner’s performance actions through actuator control. Several examples have been developed specifically for the saxophone, the target instrument of this study.

Kurosawa et al. developed a saxophone performance-support device equipped with a fingering module and its control system, along with a sensor-based mouthpiece [1]. The fingering module uses solenoid actuators, and key actuation is performed by opening and closing the keys via wire mechanisms. Based on breath-pressure input acquired by a sensor attached to the mouthpiece, the fingering control system determines the appropriate fingering actions.

Kato et al. proposed a concept for a blowing-assist device designed for performers with respiratory difficulties [2]. The device functions as a master–slave pneumatic amplifier. Two mouthpieces are used: when the performer blows into the master mouthpiece, the amplified air pressure is delivered to the slave mouthpiece.

#### 1.3.2. Indirect-Assistance Devices

Next, we present examples of indirect-assistance devices, which support learners by providing feedback on performance-related information. The feedback content and modality vary across devices. According to a review of Accessible Digital Musical Instruments (ADMIs) [6], most ADMIs that provide bimodal feedback combine auditory and visual information and include interactive GUIs or on-screen animations. Some ADMIs instead provide vibrotactile feedback in addition to auditory cues.

Matthias et al. proposed a tool for analyzing and evaluating saxophone performance [7]. Focusing on long tones—an element strongly correlated with performers’ technical level—they evaluated sustained tones and tones involving crescendo or decrescendo. The evaluation is based on frame-wise acoustic features (pitch and intensity) extracted from the performance sound. Their feedback GUI presents temporal trajectories of these acoustic features.

Molero et al. developed a piano-learning support system based on mixed reality and gamification [10]. Through MR goggles, performance cues such as the next note to be played are overlaid onto the real keyboard. Gamification elements—including scores, levels, and badges—are incorporated to encourage sustained practice.

#### 1.3.3. Devices That Combine Direct and Indirect Support

Although relatively few studies address both direct and indirect assistance, some examples exist. Chin et al. proposed a computer-assisted, multimodal performance-support system for recorder players [5]. The system provides multimodal feedback comprising auditory (performance sound), visual, and tactile cues. Tactile feedback is delivered via a C-clamp attached to the recorder, supporting the performer physically, while visual feedback presents musical notation and indicates whether fingering and blowing actions are performed correctly.

#### 1.3.4. Performer Habits Analysis

Several studies have analyzed performers’ habits; however, they mostly address performer identification rather than providing feedback to the performer. Rafee et al. analyzed multiple performances of the same piece by pianists and proposed a model that identifies performers based on expressive features such as tempo, timing, and onset deviations [11]. From the viewpoint of performer identification, the fusion of timing- and intensity-related features yielded the best results. Gindras et al. proposed a model that quantifies individual performance traits across different pieces, genres, and performance styles [12].

### 1.4. Position of This Study

**Figure 2:**
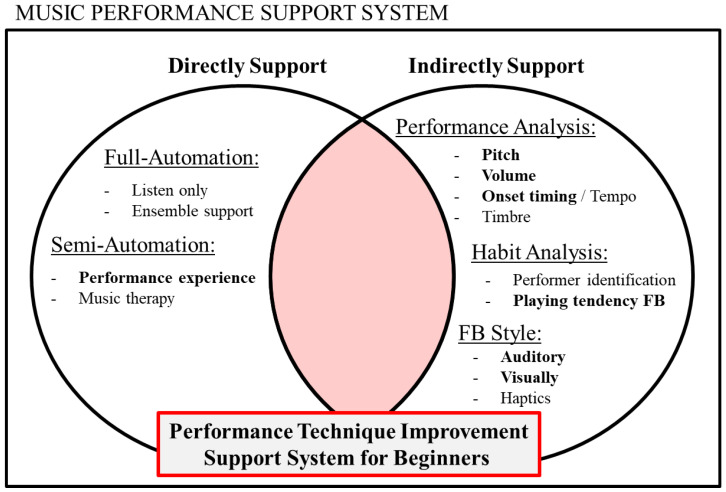
The position of this study. This figure shows that this study is positioned in the integration of direct and indirect support (red area). The underlines in the figure indicate the major support approaches, and the bold text indicates the support elements addressed in this study.

Figure 2 summarizes the position of this study within the broader context of research on instrument-performance support. Most prior studies on instrument-learning support devices have primarily focused on either direct assistance or indirect assistance alone. In contrast, this study aims to design a support device that integrates both forms of assistance, enabling complete beginners with no prior performance experience to acquire instrumental skills in a game-like and engaging manner.

As direct assistance, we automate fingering actions using a semi-automatic instrument-performance robot, as described in Section 1.1. As indirect assistance, we conduct real-time performance analysis and performer-habits analysis based on the results of the real-time analysis.

Although a multimodal recorder-support system that combines direct and indirect assistance has been reported (Section 1.3.3), the feedback in that system relies solely on sensor data attached to the fingering and blowing mechanisms; it does not evaluate the performer’s actual produced sound.

Given this limitation, novice learners—whose ability to assess their own performance is inherently limited—are particularly disadvantaged. The goal of this study is therefore to develop a system that overcomes this issue by providing sound-based evaluation and its feedback.

### 1.5. Effects of This Study

The proposed system is expected to provide the following benefits to learners:

1. Lowering the barrier of learning: The system can be used even by learners who have not yet memorized fingering patterns. Once learners acquire only minimal blowing technique, they can begin practicing through the system;
2. Improving the efficiency of skill acquisition: Learners receive real-time acoustic information feedback, enabling them to efficiently refine their performance;
3. Understanding and improving performance habits: Learners can review performance habits extracted from their past playing data. By referring to this information, they can continuously monitor and improve their instrumental skills.

## 2. Proposed System

This chapter presents the technical details of the system proposed in this study. Section 2.1 provides an overview of the system architecture, Section 2.2 describes the performance–analysis methods incorporated into the system, Section 2.3 explains the newly introduced performer-habit analysis, Section 2.4 discusses the graphical user interface (GUI) through which the system interacts with learners, and Section 2.5 details the system design for synchronizing indirect assistance (performance-information feedback) with direct assistance (semi-autonomous musical-instrument robot operation).

### 2.1. Overview

This section outlines the overall structure of the proposed system, which consists of hardware and software components. Section 2.1.1 describes the hardware configuration, Section 2.1.2 describes the software configuration, and Section 2.1.3 describes the end-to-end latency of the proposed system.

#### 2.1.1. Hardware Components

**Figure 3:**
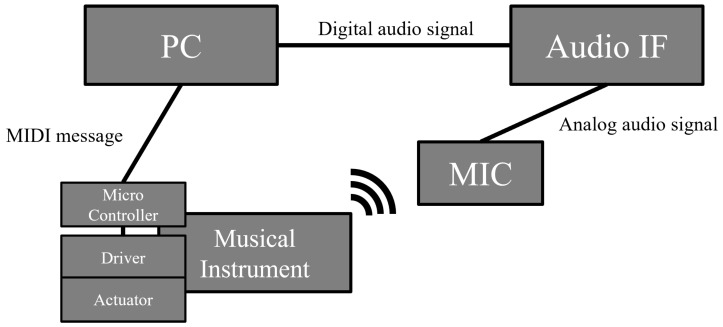
Hardware components of proposed system. The system consists of a PC, a musical-instrument robot, a microphone, and an audio interface, with the PC playing the central role in performing various processes in real time. The processes performed by the PC are shown in Figure 5.

**Figure 4:**
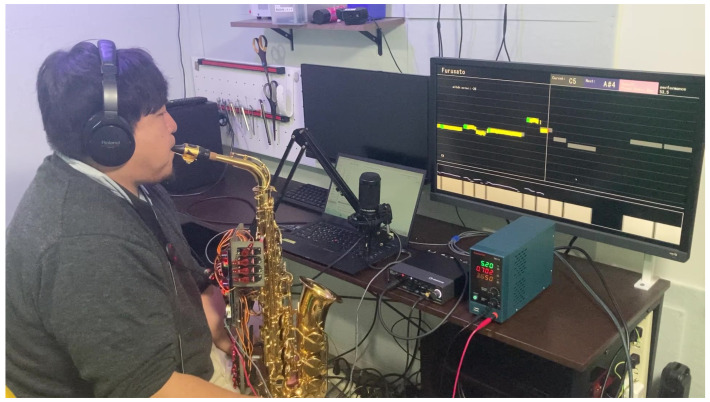
Use case of proposed system by a user. Users can receive feedback on their performance through the PC display.

Figure 3 illustrates the hardware components used in the proposed system, and Figure 4 shows an example of actual usage. Additionally, a video of actual usage is available in Supplementary Materials. The system comprises a PC, a musical-instrument robot attached to the instrument, an audio interface (Audio IF), and a microphone. In the system, the PC controls the playing actions of the musical-instrument robot, while the microphone signal is converted into digital audio by the Audio IF and subsequently delivered to the PC via ASIO, a low-latency audio-transfer driver. Therefore, an ASIO driver must be installed on the PC in advance; in this study, we employ the Yamaha Steinberg USB Driver.

While the system is normally designed to operate with the robot-augmented instrument, it can also be used with the instrument alone, without the robot. This design allows learners to gradually transition away from system assistance once their blowing technique has sufficiently improved and integrated training of fingering and blowing becomes necessary.

#### 2.1.2. Software Components

**Figure 5:**
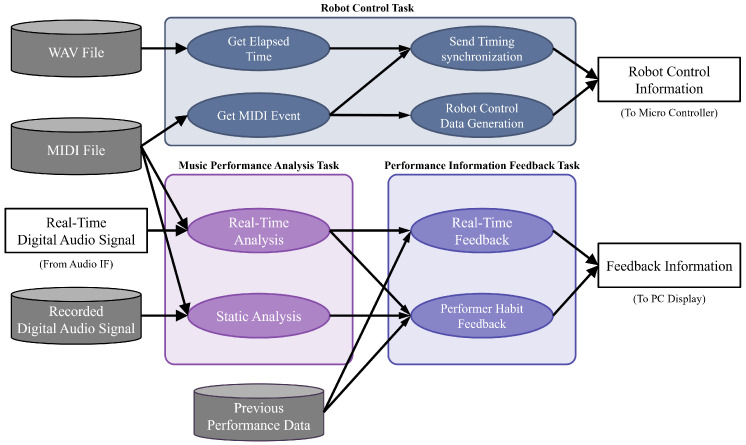
Software architecture of the proposed system, consisting of three components: Music performance analysis, performance feedback, and robot control.

Figure 5 illustrates the software components of the proposed system. The software consists of three major tasks: analysis, feedback, and communication.

The analysis task extracts acoustic features—such as pitch, loudness, and onset—from the instrument-performance audio. This includes both real-time performance analysis and offline analysis applied to pre-recorded data. The extracted features are compared with reference data, and their deviations are measured. Musical scores in the form of MIDI data are used as the performance reference. MIDI (Musical Instrument Digital Interface) is a unified standard that enables efficient communication of performance information across instruments and computers regardless of manufacturer or model [13]. The measured results are accumulated for each MIDI note and used for learner feedback. The details of the analysis task are described in Section 2.2 and Section 2.3.

The feedback task aggregates information obtained from the analysis task—including acoustic features and song information—and presents it to the learner by rendering it on the PC display. The main contents include real-time performance analysis results, score-based information such as correct pitch sequences, and other visual feedback. Details of this task are provided in Section 2.4.

The communication task sends control signals to the musical-instrument robot at appropriate timings determined from the MIDI data of the target musical piece. The transmission timing is based on the MIDI note-on events of the piece, with a predefined latency taken into account. Using the received MIDI messages, the robot’s microcontroller drives the actuators responsible for fingering actions. Details of the communication task are provided in Section 2.5.

#### 2.1.3. System Latency

Here, we will discuss the end-to-end latency of the proposed system. First, we will describe the software latency that can be measured within the proposed system’s program. In this paper, software latency is defined as the time from obtaining audio data from the ASIO buffer, through audio analysis, to completing the execution of the drawing process. As the main loop of the system runs at 60 fps, the frame time (approximately 17 ms) is the software latency deadline. Simple measurements have shown that the software latency of the proposed system is a maximum of approximately 6 ms.

The end-to-end latency additionally includes the audio input latency introduced by the audio interface and ASIO driver. According to the settings of the Yamaha USB ASIO Driver used in our setup, this input latency is approximately 50 ms.

Consequently, the total end-to-end latency of the proposed system is approximately 56 ms. The above results are summarized in Table 1.

### 2.2. Music Performance Analysis

In this section, we describe the music performance analysis (MPA) implemented in the proposed system. In this study, we selected pitch, velocity, and timing as the acoustic features to be fed back to the performer. These features were chosen with reference to the related studies discussed in Section 1.3, focusing on those that are suitable for real-time analysis.

In the real-time analysis task, the audio stream is processed on a frame-by-frame basis, where one frame consists of 2048 samples (approximately 46 [ms]) at a sampling rate of 44.1 [kHz]. For the extraction of each acoustic feature, algorithms that achieve high speed and high accuracy even with short frame lengths are employed. The following terms describe the extraction methods for each feature and the procedures for presenting the extracted information as feedback.

#### 2.2.1. Pitch

In music acoustics, pitch is one of the three primary attributes of sound and represents a fundamental feature for performance analysis. Pitch estimation corresponds to estimating the fundamental frequency (f0) of a performed sound signal, and numerous algorithms have been proposed for this purpose.

In the proposed system, we adopt DIO [14], included in the WORLD (C++, GitHub version based on v1.0.0) speech-synthesis toolkit [15], as the real-time pitch estimation algorithm because it provides both high accuracy and sufficient computational efficiency for real-time execution. DIO exploits the harmonic structure of the input signal: it passes the signal through multiple low-pass filters (LPF) with different cutoff frequencies, computes several f0 candidates and their reliability scores from each filtered signal, and finally selects the candidate with the highest reliability as the estimated f0.

**Figure 6:**
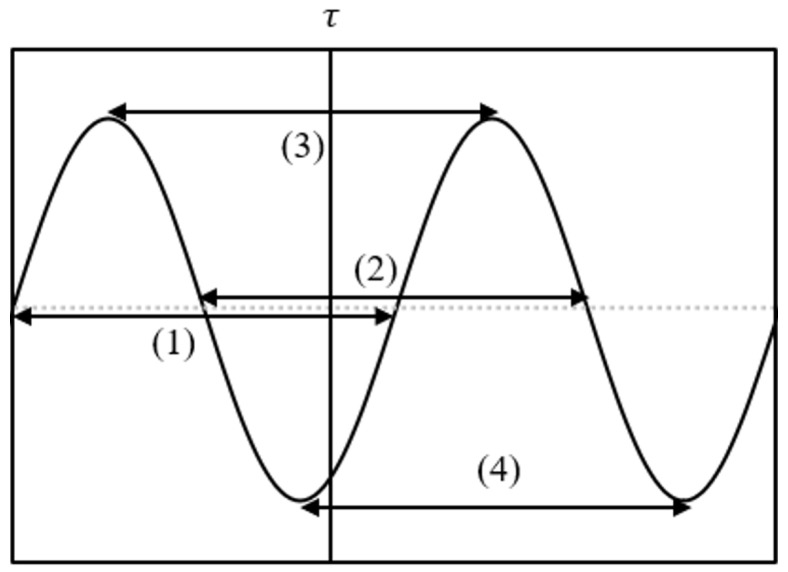
Dio’s f0 candidate estimation. DIO calculates four periods represented by (1)–(4) in the figure. (1) is the period between zero crossings going from negative to positive, (2) is the period between zero crossings going from positive to negative, (3) is the period between positive peaks, and (4) is the period between negative peaks.

This section describes how DIO computes f0 candidates and their reliability from LPF-processed signals. As illustrated in Figure 6, DIO evaluates four types of periodicity intervals, including zero-crossing and peak-to-peak intervals. When higher harmonics are suppressed by LPFs and the fundamental component is emphasized, the waveform more closely resembles a sinusoid. Therefore, DIO uses the inverse of the averaged intervals as an f0 candidate and uses the standard deviation of these intervals as its reliability measure. A smaller standard deviation indicates a higher confidence in the candidate.

DIO provides parameters for setting the lower and upper bounds of the f0 search range. In this study, we set the lower bound to 100 [Hz] to cover D♯3, the lowest pitch of the alto saxophone.

Furthermore, the proposed system refines the f0 values estimated by DIO using StoneMask [15], an instantaneous frequency-based correction algorithm also implemented in WORLD. In StoneMask, the instantaneous frequency is computed using Flanagan’s equation [16], as shown Equations (1)–(3), where w(t) denotes the window function used for analysis and x(t) denotes the input signal.

(1) $$\begin{array}{cc} \omega_{i}\left(\omega ,t\right) & =\frac{R\left[S\left(\omega ,t\right)\right]I\left[\frac{dS(\omega ,t)}{dt}\right]-I\left[S\left(\omega ,t\right)\right]R\left[\frac{dS(\omega ,t)}{dt}\right]}{{\left|S\left(\omega ,t\right)\right|}^{2}}, \end{array}$$

(2) $$\begin{array}{cc} S(\omega ,t) & =\int w\left(\tau -t\right)x\left(\tau \right)e^{-j\omega \tau }d\tau , \end{array}$$

(3) $$\begin{array}{cc} \frac{dS(\omega ,t)}{dt} & =\int \frac{dw(\tau -t)}{dt}x\left(\tau \right)e^{-j\omega \tau }d\tau . \end{array}$$

For offline performance analysis, we use HARVEST [17], which is likewise included in WORLD. Although HARVEST is computationally slower than DIO, it provides superior estimation accuracy and robustness against noise.

The estimated f0 values obtained through the above procedure are compared with the pitch information in the reference MIDI data. Specifically, the estimated f0 is converted into a real-valued MIDI note number, and the pitch error relative to the correct 12-tone equal temperament MIDI note is computed in cents (where one semitone equals 100 cents). These error values are aggregated for each reference MIDI note, from which the mean error (ME), mean absolute error (MAE), and standard deviation (SD) are calculated. The resulting evaluating information is used both for post-performance learner feedback and for performer-habit analysis.

The pitch estimation precision of DIO and HARVEST was verified through a simple evaluation using synthetic harmonic signals under conditions matching those used in this study, confirming that both tools exhibit sufficiently high precision for performance analysis. Specifically, the mean absolute error between the estimated F0 and reference frequency was approximately 0.094 cents for DIO and 0.081 cents for HARVEST, which are well below perceptual discrimination thresholds.

#### 2.2.2. Velocity

In this system, we adopt the LUFS (Loudness Units relative to Full Scale) metric for loudness measurement. LUFS is a perceptually motivated loudness measure standardized in ITU-R BS.1770 [18] and EBU R128 [19], which reflects human auditory sensitivity by applying frequency weighting. Unlike dBFS, which represents raw signal amplitude, LUFS provides a perceptually more relevant representation of perceived loudness.

In the proposed system, loudness is computed on a frame-wise basis (400 [ms], 100 [ms] overlap) using instantaneous LUFS values, without long-term temporal integration. Specifically, the input signal is first processed by a K-weighting filter, and the loudness of each analysis frame is calculated from the mean squared energy of the weighted signal. The instantaneous LUFS value used in this study is defined as shown in Equation (4), where $E_{signal}$ denotes the frame-wise mean squared energy after K-weighting.

(4) $$LUFS=-0.691+10\log_{10}\left(E_{signal}\right)$$

Although loudness can be estimated using the above procedure, loudness estimation differs substantially from pitch estimation in one key respect: it is highly sensitive to recording conditions such as microphone placement and the acoustic properties of the recording environment. To address this dependency, the system measures the loudness characteristics of the performance environment immediately before each session and uses them to derive a scaling function between LUFS-based loudness and MIDI velocity.

Three types of reference audio signals are acquired from the instrument:

1. maximum performance loudness (${LUFS}_{max}$);
2. minimum performance loudness (${LUFS}_{min}$);
3. background (ambient) noise level (${LUFS}_{mute}$).

For each signal, its LUFS value is computed, and the threshold LUFS for detecting voiced frames is defined as the average of the minimum-performance LUFS (${LUFS}_{min}$) and ambient-noise LUFS (${LUFS}_{mute}$). During actual musical performance, analysis is performed only on frames whose LUFS exceeds ${LUFS}_{th}$. The range [${LUFS}_{th}$, ${LUFS}_{max}$] is then divided into 128 segments and linearly mapped to MIDI velocity values in the range [0, 127]. The difference between the estimated velocity and the reference MIDI velocity is computed and fed back to the performer. If the measured LUFS exceeds ${LUFS}_{max}$, the velocity is clipped to the maximum value of 127.

#### 2.2.3. Onset Timing

According to a survey on Music Performance Analysis (MPA) [20], pronunciation timing is identified as one of the key evaluation metrics in performance analysis. In alto saxophone playing, determining the sharp attack produced by tonguing is also beneficial. Therefore, in this system, performance timing is analyzed by estimating the onset timing of the performance signal and computing its deviation from the onset times of consecutive ground-truth MIDI notes. As the onset detection algorithm, the system adopts the complex-domain method [21]. The onset detection function η is defined according to the complex-domain formulation proposed in [21], as shown in Equations (5) and (6).

(5) $$\begin{array}{cc} \eta (m) & =\Sigma_{k=1}^{K}\Gamma_{k}\left(m\right), \end{array}$$

(6) $$\begin{array}{cc} \Gamma_{k}\left(m\right) & ={\left\{{\left[R\left({\hat{S}}_{k}\left(m\right)\right)-R\left(S_{k}\left(m\right)\right)\right]}^{2}+{\left[I\left({\hat{S}}_{k}\left(m\right)\right)-I\left(S_{k}\left(m\right)\right)\right]}^{2}\right\}}^{\frac{1}{2}}. \end{array}$$

The complex-domain method measures changes in the frequency spectrum across successive FFT frames and recognizes frames that exhibit a local temporal maximum in this change as onsets. A distinctive feature of this method is that it considers variations not only in the amplitude spectrum but also in the phase spectrum. The detection function is defined as the sum of Euclidean distances between two complex numbers representing amplitude–phase information in the current and previous FFT frames.

In Equation (5), *m* denotes the input frame index and *k* denotes the FFT-bin index. $S_{k}$ represents the complex value obtained from the *m*-th input frame, whereas ${\hat{S}}_{k}$ represents the complex value obtained from a past input frame.

Each detected onset is assigned to exactly one ground-truth MIDI note. To prevent excessive onset detections, the detection interval is restricted to a temporal window of $\pm T_{\mathrm{onset}}$ [ms] around the ground-truth note-on time of each MIDI note. In this study, $T_{\mathrm{onset}}$ is set to one-fourth of each note duration.

For evaluation, the temporal difference [ms] between each detected onset and the note-on time of the corresponding MIDI note is used. If no onset is detected within $\pm T_{\mathrm{onset}}$ of a MIDI note’s note-on time, the system determines that no (sharp) onset occurred.

### 2.3. Performer’s Habits Analysis

In this section, we describe the Performer’s Habits Analysis implemented in the proposed system. This study proposes a pitch-accuracy-focused method that provides feedback on whether a learner tends to struggle with the performance actions required for transitions from pitch state $S_{i}$ to $S_{j}$.

This functionality enables the system to present habit information that learners may not easily be aware of on their own, such as difficulties in specific fingerings, embouchure configurations, or changes between these performance actions. The basic concept of the habits-analysis method is described in Section 2.3.1, and the handling of past performance data used for habits analysis is presented in Section 2.3.2.

#### 2.3.1. Habits Analysis Methods

We assume the following two factors as “performer’s habits”:

- When performing a particular single pitch, the learner tends to produce a pitch that is systematically higher (or lower) than the target;
- When performing a pitch following a particular pitch transition, the learner tends to produce a pitch that is systematically higher (or lower) than the target.

A habit originating solely from a single pitch may indicate that the learner simply struggles with the performance action required for that pitch. In such cases, habit analysis can be performed by collecting performance-analysis results for each pitch independently. A habit arising from pitch transitions, on the other hand, is considered to be caused by the learner’s difficulty in executing the change in performance action associated with the transition.

In the proposed habit analysis, transitions between adjacent notes are defined based on their temporal continuity. Note pairs separated by an interval corresponding to a quarter rest or longer, calculated with respect to the song’s tempo, are excluded from transition analysis.

#### 2.3.2. Analysed Data Storage

A sufficiently large amount of performance data are required to analyze a performer’s habits. Therefore, the proposed system accumulates past performance data and uses them for habit analysis. In addition to evaluation data obtained during real-time performance using the system, evaluation data derived from audio recordings obtained without the system are also utilized.

To enable these functions, we propose a method that incorporates real-time performance data obtained using the system and offline performance data obtained without the system into the same Markov model without distinction. Specifically, structured evaluation data are stored in the system in JSON format, and JSON files containing real-time evaluation data, past evaluation data, and offline evaluation data are merged. The integrated JSON file is referenced during habits analysis, and the aggregated data are stored as the updated past-data JSON file for use in subsequent performance sessions.

### 2.4. GUI

In this section, we describe the graphical user interface (GUI) of the proposed system, which not only provides the analytical results presented in Section 2.2 and Section 2.3 as feedback but also offers various types of information to learners. Hereafter, the GUI of the proposed system is simply referred to as the GUI. The GUI was developed using the DX Library, a multimedia library for C++.

The GUI consists of the following three steps. The details of each step are described below.

Step 1. Prepare;
Step 2. Performance;
Step 3. Result display.

#### 2.4.1. Prepare

**Figure 7:**
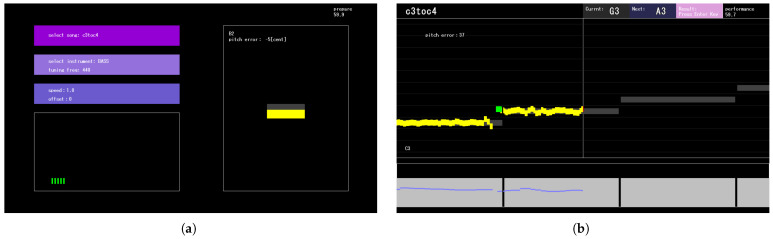
GUI during preparation and musical performance. (**a**) GUI during performance preparation. The top-left area allows the learner to configure performance parameters, the bottom-left area provides controls for measuring performance loudness, and the right area allows tuning. (**b**) GUI during musical performance. The center displays pitch and timing information. The target pitch is drawn in dark gray, the pitch estimated from the performed audio signal is drawn in yellow, and the estimated onset is drawn in green. The bottom area presents loudness information. The target volume is drawn in light grey, and the estimated volume in blue.

In the first step, the learner configures system parameters and prepares for performance through environment measurement and tuning. An example of the GUI during this preparation stage is shown in Figure 7a.

The upper-left area provides parameter settings such as the musical piece, the instrument used, and the offset. The offset parameter adjusts the temporal alignment between the system’s visual rendering and the instrument robot’s performance timing. Details regarding the synchronization between the system and the instrument robot are described in Section 2.5.

The lower-left area instructs the learner to measure the performance environment. This measurement corresponds to acquiring the loudness reference values described in Section 2.2.2.

On the right side, the GUI provides a pitch-feedback feature modeled after conventional instrument tuners. Learners can tune their instruments by referring to this area. As noted in Section 2.2.1, the DIO algorithm is used for pitch estimation in the tuner.

#### 2.4.2. Performance

During the performance step, the system controls the instrument robot, performs real-time music analysis, and provides feedback to the learner.

The real-time analysis method is as described in Section 2.2. Here, we focus on how real-time analysis results are presented through the GUI. An example GUI display during musical performance is shown in Figure 7b. The upper area displays system messages, the title of the musical piece, the current performed pitch, and the next pitch to be played. In the central area, the ground-truth pitch is displayed in gray and the estimated pitch in yellow, enabling visual comparison. A vertical line in the center acts as the judge line, and the ground-truth pitch overlapping this line corresponds to the current note. When an onset is detected, the estimated pitch is highlighted in green to indicate the performance onset timing. In the lower area, the ground-truth velocity is displayed in light gray, while the estimated velocity is shown in blue, enabling visual comparison between the two in the same manner as the pitch information.

#### 2.4.3. Result Display

After the performance, the system presents the evaluation results to the learner. The result-display interface provides an overview of the performance, detailed evaluations for individual notes, and the results of habits analysis.

**Figure 8:**
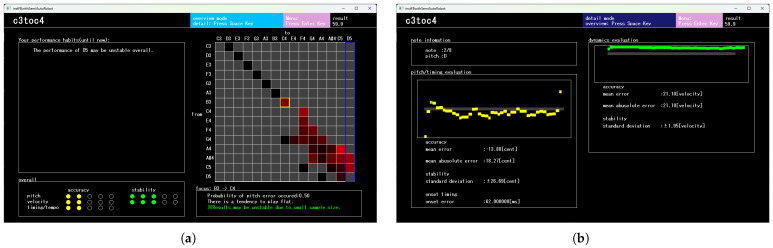
GUI after the musical performance. (**a**) The lower-left area presents the overall performance score for the piece, evaluated on a five-point scale for each performance element and color-coded according to the rating (1: red, 2: yellow, 3 or higher: green).The upper-right area shows a transition-matrix heatmap derived from the habit analysis. Individual cells in the heatmap are selectable, and the corresponding evaluation details for the selected cell are displayed in the lower-right area.The upper-left area provides feedback indicating difficulty in producing a specific pitch, which is highlighted by a blue rectangle in the heatmap. (**b**) Detailed results for each note. For each note, the learner can review the performance trajectory and evaluation metrics for each acoustic feature. The upper left area shows basic information about the note, the lower right area shows evaluation information about pitch and onset, and the upper right area shows evaluation information about volume.

Figure 8 shows the GUI used to present overall performance results and habits-analysis outcomes. The lower-left area presents a five-level evaluation of the performance. The evaluation metrics, accuracy and stability, are defined as follows: accuracy reflects the correctness of each acoustic feature and is quantified using the MAE, while stability reflects the steadiness of each feature and is quantified using the SD. The right area presents the transition matrix from the habits analysis as a heatmap. In this heatmap, transitions with high error rates $P_{err}\left(i,j\right)$ are shown in red, and those with low error rates are shown in black. Transitions not observed in past performances are shown in gray.

As described in Section 2.3.1, if the values of $P_{err}\left(i,j\right)$ are consistently high for transitions leading to a particular pitch state $S_{j}$, it suggests that the learner may struggle with producing the pitch corresponding to $S_{j}$ itself rather than with specific transitions. In such cases, as shown in Figure 8a, the system highlights the corresponding column in the transition-matrix heatmap with a blue frame.

Moreover, during the result-display step, the learner can switch to a more detailed view showing evaluations for each note. Figure 8b provides an example. For pitch and loudness evaluation, detailed feedback is given by presenting both the performance trajectories and numerical indicators of accuracy and stability. For timing evaluation, the system presents the error—measured in milliseconds—relative to the ground-truth timing defined by the MIDI note-on event.

Through these visualization and feedback functions, the proposed system supports efficient and effective skill improvement for learners.

### 2.5. Semi-Automatic Musical Instruments Robot and Its Control

In this section, we provide an overview of the semi-automatic alto saxophone performance robot that operates in synchrony with the proposed performance-analysis and feedback system, as well as the method used to synchronize the robot with the system. For details regarding the mechanical structure of the instrument robot, readers are referred to [4].

The performance control of the instrument robot is carried out via MIDI messages. The transmitted MIDI messages primarily contain the note-on flag and information regarding the performance pitch. By sending these MIDI messages from the system to the robot with appropriate timing, the system synchronizes the robot’s performance actions (i.e., fingering motions) with the ground-truth MIDI notes defined within the system. Communication of MIDI messages between the system and the robot is achieved through USB-MIDI transmission using a wired USB connection [22].

To implement those synchronizations, the system utilizes musical pieces in WAV format. A musical audio file is prepared in advance so that its performance strictly follows the ground-truth MIDI data. When the system transitions to the performance screen described in Section 2.4.2, the audio file begins playback. During playback, the system continuously monitors the current playback time. Whenever the playback time surpasses the onset time of a MIDI note included in the ground-truth MIDI data, the system sends the corresponding note-on MIDI message to the instrument robot.

## 3. Evaluation Experiment

In this chapter, we describe three experiments conducted to examine whether the proposed system’s performance analysis and feedback functionalities contribute to improving learners’ musical performance. An overview of each experiment is provided below.

Experiment 1 (Exp. 1). Interviews with trained saxophone players (pilot study);
Experiment 2 (Exp. 2). Evaluation of the system’s ability to support performance improvement for novice saxophone learners;
Experiment 3 (Exp. 3). Evaluation of the system’s habit-analysis functionality.

**Figure 9:**
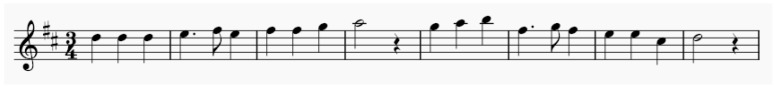
A part of the score for “Furusato”.

In all experiments, we used the song “Furusato,” composed by Teiichi Okano, as the performance piece. This piece was selected because it is widely familiar to participants, has a moderate tempo (BPM 84), and is considered relatively easy for beginners to perform with stability. Basic information about the piece is shown in Table 2, and the musical score for the first eight measures is presented in Figure 9. The alto saxophone is an E♭ transposing instrument; therefore, the score in Figure 9 is written in D major, corresponding to F major in concert pitch. In the MIDI data for “Furusato,” the velocity value was fixed at 80.

All experiments were conducted under identical environments. The main experimental environment is summarized in Table 3.

### 3.1. Experiment Methods

#### 3.1.1. Exp. 1—Pilot Study: Interviews with Trained Saxophone Players

The aim of Experiment 1 was to investigate whether the proposed system can potentially contribute to improving musical performance skills from the viewpoint of trained instrumental players. One participant with saxophone performance experience took part in the experiment. The participant was a male in his twenties with a total of six years of saxophone-playing experience. Note that this participant was not involved in the system design of this study.

The experiment followed a walk-through format, in which the participant used the system while receiving explanations of its purpose and functionality. During system use, the participant performed the target piece multiple times. After the performances, a post-task interview regarding the system was conducted.

#### 3.1.2. Exp. 2—Evaluation of the System’s Support for Improving Novice Performance

The aim of Experiment 2 was to examine whether the proposed system’s real-time performance-analysis and feedback functions could support performance improvement among novice saxophone learners. 11 novice participants took part in the experiment. Although all participants were beginners with respect to saxophone performance, The participant’s musical instrument playing experience can be divided into three main types:

1. Those with experience playing wind instruments other than saxophone (2 persons);
2. Those with experience playing instruments other than wind instruments (4 persons);
3. Those with no experience playing instruments (5 persons).

Experiment 2 proceeded according to the following steps:

Procedure 2-1. Provide participants with a preliminary explanation of the purpose and overview of the proposed system;
Procedure 2-2. Have participants alternately perform using the proposed system and without using it, and record the performance audio in both conditions;
Procedure 2-3. Repeat Step 2 five times;
Procedure 2-4. Perform static analysis on the recorded audio of both conditions and compare the analysis results.

For the “performance without the proposed system,” a MIDI sequencer was used. The MIDI sequencer displays MIDI notes in a piano-roll format and, as in the proposed system, enables performance of the semi-automatic saxophone robot via MIDI messages. In this paper, we used Domino by TAKABO SOFT as the MIDI sequencer. Regardless of performance condition, audio recordings were made using Audacity.

Details of the evaluation method for performance audio are provided in Section 3.2.

#### 3.1.3. Exp. 3—Evaluation of the Habit-Analysis Function

The aim of Experiment 3 was to qualitatively evaluate how the proposed habit-analysis function provides feedback to learners and whether the information it outputs can contribute to improving their performance.

Unlike Experiment 2, which quantitatively evaluates pitch accuracy using MAE and statistical testing, this analysis is exploratory and illustrative in nature.

In this experiment, the performance data obtained in Experiment 2 is used as input. Therefore, the number of participants and their musical instrument playing experience are the same as in Experiment 2.

Experiment 3 proceeded according to the following steps:

Procedure 3-1. Record 5 performances with the proposed system and 5 performances without the proposed system;
Procedure 3-2. Perform static analysis on all recorded performance audio and generate JSON-format habit analysis;
Procedure 3-3. Input the habit analysis into the proposed system and generate a transition matrix;
Procedure 3-4. Examine the generated transition matrix and discuss the performer’s habits.

### 3.2. Evaluation Criteria

This section describes the static audio-analysis methods and evaluation criteria used in Experiments 2 and 3.

As the static analysis method, we performed f0 estimation using HARVEST, as described in Section 2.2.1. Based on the estimated f0, we computed the deviation from the ground-truth MIDI notes in units of cents and calculated the MAE, ME, and standard deviation (SD).

In Experiment 2, the MAE between the two performance conditions was compared to assess pitch accuracy, the ME was compared to examine bias tendencies in performance errors, and the SD was compared to evaluate performance stability. All metrics were calculated using the average of five performances, rounded to the nearest third decimal place. In addition, we conducted a paired *t*-test on the MAEs obtained from the two types of performance. In this paper, we conducted a one-sided test with a significance level of α=0.05.

Because the semi-automatic saxophone robot mechanically replaces fingering operations, mismatches can occur between the device’s fingering transitions and the performer’s blowing timing, occasionally producing unintended sounds. To prevent such anomalous data from disproportionately influencing the evaluation, we excluded frames whose f0 deviation from the ground-truth MIDI note exceeded 600 cents (0.5 octaves) when computing evaluation metrics for Experiment 2.

In Experiment 3, a transition matrix was constructed based on these evaluation metrics, and its content was analyzed. For the probability parameter $P_{err}\left(i,j\right)$ in the transition matrix, the MAE threshold was set to 50 cents.

This threshold was selected as a design criterion for identifying pitch-transition errors. In a 12-tone equal temperament system, a deviation exceeding ±50 cents causes the nearest pitch category to change, which can be interpreted as playing an incorrect musical note rather than a slight intonation deviation.

### 3.3. Results

The interview findings from the pilot study in Experiment 1 are presented in Section 4. In this section, we report the experimental results obtained in Experiments 2 and 3.

For Experiment 2, Table 4 summarizes the average MAE, ME, and SD values computed over the entire performance for both conditions—using the proposed system and not using it.

**Figure 10:**
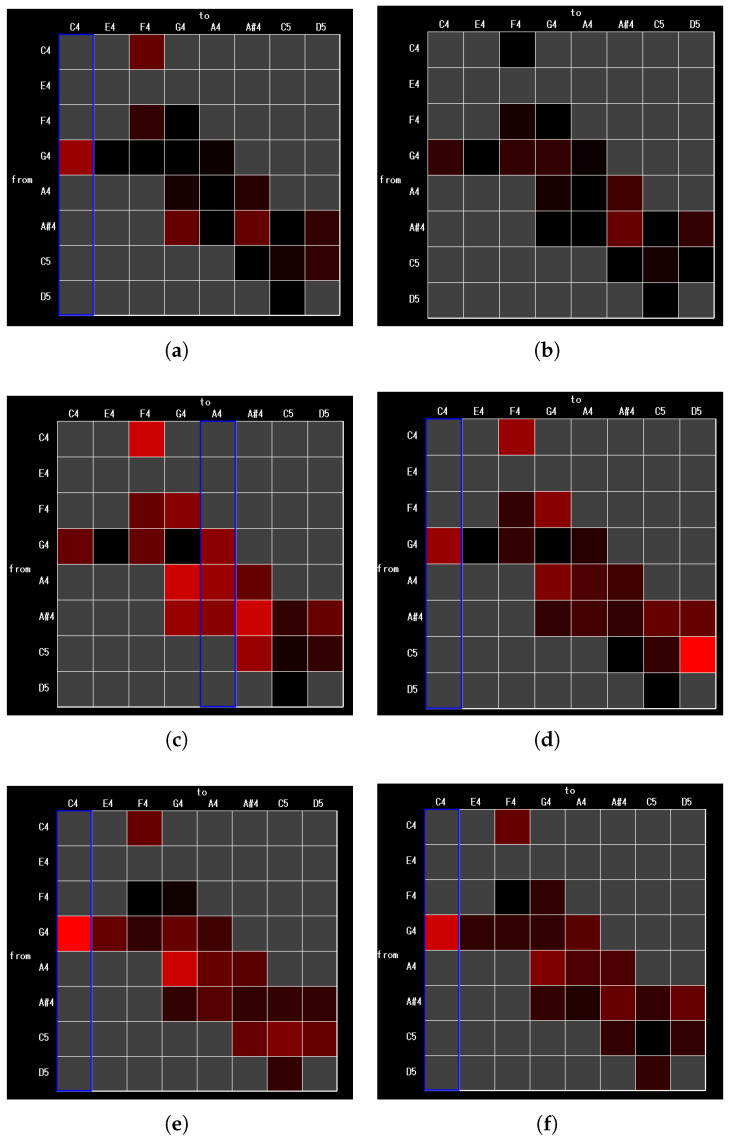
Comparison of transition matrices in Experiment 3. For each pitch transition in the piece, the heatmap visualizes the proportion $P_{err}\left(i,j\right)$ of transitions for which the performed pitch exhibits an MAE of 50 [cent] or more. Cells shaded red indicate higher values of $P_{err}\left(i,j\right)$, while darker cells indicate lower values. Gray cells denote transitions that do not occur in the piece. Panels (**a**,**b**), (**c**,**d**), and (**e**,**f**) show the transition matrices for participants B, D, and I, respectively, without and with the proposed system. When high error rates are consistently associated with a specific pitch state $S_{j}$, the corresponding column in the transition matrix is highlighted with a blue frame, indicating a habitual difficulty in producing that pitch.

For Experiment 3, of the 11 participants in the experiment, the transition matrices for three participants are shown in Figure 10: participant B, who has experience playing wind instruments other than the saxophone; participant D, who has experience playing instruments other than wind instruments; and participant I, who has no experience playing an instrument. The numbering of the subjects corresponds to Table 4.

## 4. Discussion

To clarify the motivation and expected benefits of the proposed approach, we first discuss the differences between performances with and without the proposed system before addressing the experimental results.

In a conventional learning setting without support, novice saxophone players are required to memorize fingering patterns while simultaneously reproducing the musical score and controlling sound production. This multitasking imposes a substantial cognitive load, making it difficult to focus on embouchure and airflow control during the early learning stage.

By contrast, the proposed system delegates the fingering task to a semi-automated instrument-playing robot, allowing learners to concentrate primarily on sound production. Once a minimal requirement—producing sound with a mouthpiece—is satisfied, learners can immediately practice blowing control using actual musical pieces as training material. This task separation lowers the initial learning barrier and facilitates focused training of sound production without requiring attention to fingering details.

### 4.1. Exp. 1—Pilot Study: Interviews with Trained Saxophone Players

Necessity of the system. Before conducting the system-based performance session, we asked the participant about the perceived need for the proposed system. According to the interview responses, the participant reported that they modify their embouchure and other performance actions depending on the pitch being played. In particular, they consciously adjust the airflow velocity and the pressure of the lips on the mouthpiece. These observations indicate that the participant finely regulates their playing gestures depending on the target pitch, suggesting that a system capable of visualizing and providing feedback on such actions would be beneficial.

System usability. The participant stated that the tuner in the preparation screen was sufficiently easy to read. During the performance, they focused on the pitch bar and assessed how far the upcoming pitch was from the current one by observing the incoming bars. Regarding onset detection, the participant reported occasional false negatives in which tongued notes were not detected as onsets, whereas false positives—onset detections at non-tongued notes—did not occur.

System effectiveness. Although the five-grade overall performance rating was visually easy to interpret, the participant noted that the habit-analysis heatmap was difficult to understand without prior explanation. Regarding the detailed result display, the participant mentioned the potential usefulness of graphical trajectory feedback. They also commented that a larger amount of performance data are necessary to evaluate the validity of the detected performance habits.

### 4.2. Exp. 2—Evaluation of the System’s Support for Improving Novice Performance

Table 4 shows that, when using the proposed system, the pitch MAE decreased for 9 out of the 11 participants. In Experiment 2, the five-trial averaged MAE values were distributed approximately between 15 and 40 cents. It has been reported that the pitch discrimination threshold of the human ear for a 1 kHz pure tone is approximately 10–15 cent [23]. This comparison suggests that, for saxophone novices, continuously adjusting pitch while performing a melody is a challenging task.

A paired *t*-test conducted on the MAE values revealed a statistically significant difference (p=0.022), leading to the rejection of the null hypothesis that the proposed system does not reduce pitch MAE compared to performances without the system. These results suggest that the proposed system has the potential to improve pitch accuracy in musical performance.

However, the effect size was relatively small (d=0.335), indicating that the magnitude of improvement is limited and may vary across individuals.

The other two metrics exhibited participant-dependent behavior. A common trend observed in the ME and SD results is that system use did not cause major deterioration but occasionally led to substantial improvement.

These results indicate that the proposed system may facilitate training that promotes greater awareness of note values in performance.

### 4.3. Exp. 3—Evaluation of the Habit-Analysis Function

Figure 10 shows representative transition matrix heatmaps obtained from three participants. Compared with the condition without the proposed system, the heatmaps obtained with the proposed system exhibit a general tendency toward reduced error occurrences across pitch transitions. As shown in Table 4, all three participants also demonstrated improved MAE values when using the proposed system. These observations are consistent with the quantitative results reported in Experiment 2, suggesting that the proposed habit analysis can serve as a visual aid that complements quantitative performance metrics by making pitch-error habits more interpretable.

Focusing on individual pitch transitions, participant B (Figure 10a,b) exhibited improved performance in low-register transitions such as G4→C4 and C4→F4 when using the proposed system. For participant D (Figure 10c,d), improvements were observed over a wide range of mid-register transitions, whereas relatively large errors remained in a specific high-register transition (C5→D5). Participant I (Figure 10e,f) showed no substantial overall change, but a slight improvement trend was observed from the mid to the high register.

These results indicate that providing learners with cumulative, visually interpretable feedback on their performance may help them recognize recurring pitch-transition errors, i.e., performance habits, and support self-directed improvement. In addition, a practical advantage of the proposed system is that transition matrices can be generated not only from real-time performances but also from externally recorded audio, enabling retrospective analysis and reflection.

On the other hand, while the present study discusses the potential of the habit analysis framework as a supportive visualization tool, it does not verify whether continued use of the system leads to long-term improvement of specific performance habits. Evaluating whether sustained practice with awareness of particular pitch transitions results in measurable behavioral changes remains an important topic for future work.

### 4.4. Limitation

Through the experiments, several limitations of the proposed system have become apparent, as summarized below.

On the timing synchronization between blowing and fingering. The current system provides timing guidance for blowing actions relative to automated fingering mainly through GUI-based instructions. However, such indirect guidance inevitably has limitations in conveying precise timing information with low cognitive load. Improving the effectiveness of timing instruction and synchronization between blowing and fingering remains an important direction for future work.

On performance feedback and the graphical user interface (GUI). Many participants primarily focused on the pitch-feedback display while using the system and paid relatively little attention to the loudness-feedback display. Similar to how timing information is integrated with the pitch-feedback view, loudness information should also be presented in an integrated manner to improve perceptual accessibility.

In addition, experienced saxophone players reported the need for a musical score. Learning to read musical notation is one of the essential steps for beginners, as it connects directly to fundamental concepts such as meter and note values. Although the proposed system is designed to allow performance without requiring score-reading skills, the current design makes it difficult to handle articulation information.

On real-time performance analysis. Real-time analysis fundamentally involves trade-offs with system responsiveness. The system uses DIO for pitch estimation; however, as DIO was originally designed for human voice, it may exhibit decreased accuracy in the higher frequency range.

For loudness analysis, because the mapping between LUFS and velocity is linear, perceived loudness may not always match the numerical values. Designing appropriate velocity curves for individual instruments may mitigate this issue.

Regarding timing analysis, the system could not detect all onsets accurately. To avoid false positives, the detection threshold was set relatively high. Consequently, the system currently functions primarily as a detector of sharp attacks, such as tonguing. A potential improvement would be to provide feedback by selecting the most plausible onset candidate within each detection window when no value exceeds the threshold.

The proposed system performed real-time analysis of basic performance elements for instrument learners. However, the current system does not evaluate advanced musical expressions such as pitch bends and ritardandos. This is because the target users of the proposed system include beginners, and the goal is to provide clear and consistent evaluation even in the early stages of performance training.

On the performance-habit analysis function. The habit-analysis function requires a sufficient amount of performance data from each learner. Therefore, providing one-shot feedback on individual performance habits is difficult. In addition, characteristics specific to the piece or the instrument are not yet separated from the performer’s individual habits. Moreover, the current implementation considers only pitch-related information, whereas other performance attributes should also be included.

### 4.5. Future Work

#### 4.5.1. Extension of Performance Analysis

In the current implementation, pitch evaluation is based on a fixed 12-tone equal temperament (12-TET) reference, which provides a clear and reproducible criterion for individual practice and monophonic performance training. However, it is well known that optimal pitch targets in wind instrument performance can depend on musical context, such as harmonic function in ensemble playing or expressive intonation adjustments.

Incorporating alternative tuning systems, including just intonation and context-dependent adaptive pitch targets, remains an important direction for future work. By integrating harmonic context or musical function into the pitch evaluation framework, the proposed system could be extended to support ensemble performance training and more advanced expressive intonation practice.

Among the performance attributes not addressed in this paper, timbre is particularly important. Several approaches have been proposed that evaluate timbre by measuring “closeness to expert performance.” Incorporating such methods could extend the range of informative feedback available to learners.

Furthermore, along with providing score information, future extensions may include articulation analysis via MIDI Control Change messages.

#### 4.5.2. Extension of Performance-Habit Analysis

This study examines a pitch-transition model based on a Markov model for habit analysis. However, the transition probabilities themselves were not used directly for feedback. One possible application, inspired by phrase generation using HMMs [24], is to automatically generate performance phrases that learners are likely to find difficult.

#### 4.5.3. Extension of Target Instruments

Although the present study focuses on the saxophone, the proposed support system is not inherently instrument-specific. For flute, semi-automated instrument-playing robots have already been developed, and the proposed system could be applied to such instruments with minimal modification.

Furthermore, for other woodwind instruments in which fingering and sound production can be clearly separated—such as the clarinet, oboe, and bassoon—the proposed system is in principle applicable once an appropriate semi-automated playing robot becomes available. In this sense, the primary limitation lies in hardware availability rather than in the software framework of the proposed system.

## 5. Conclusions

In this paper, we propose a beginner-friendly system for improving saxophone performance skills by combining direct support through a semi-automatic performance robot with indirect support through real-time performance feedback and performance-habit analysis.

By providing real-time feedback on pitch, loudness, and onset timing, the system enables learners to immediately reflect on and adjust their performance. In addition, the integration of the semi-automatic instrument-playing robot allows even inexperienced learners to engage in saxophone practice with low entry barriers.

By focusing on changes in pitch-accuracy values during pitch transitions, the system generates a transition matrix based on a Markov model—derived from accumulated performance data—to visualize “error rates” for each transition. Presenting this information helps learners recognize performance habits that are otherwise difficult to notice.

In the experiment, we conducted a pilot study targeting experienced saxophone players and measured the effectiveness of the proposed system targeting beginner saxophone players. The pilot study showed that the proposed system has the potential to contribute to supporting performance for beginners. In the experiment conducted on beginners, we confirmed that using the proposed system significantly improved the MAE of the pitch of the played notes compared to when the proposed system was not used.

Consequently, the system is expected not only to assist novice players but also to lower the barriers for complete beginners to begin learning musical performance.

## Figures and Tables

**Table 1:** End-to-end latency of the proposed system.

Component	Latency [ms]
Input Latency	≈50
Software Latency	≤6
End-to-end Latency	≈56

**Table 2:** “Furusato” information.

BPM	84
Duration [s]	38
key	D major

**Table 3:** Experiment enviroment.

Instrument	Maker	Model
Musical Instrument	YAMAHA (Hamamatsu, Japan)	YAS-280
Microphone	audio-technica (Tokyo, Japan)	AT2035
Audio Interface	steinberg (Hamburg, Germany)	UR22C
PC	Lenovo (Beijing, China)	ThinkPad X1
OS	Microsoft (Redmond, WA, USA)	Windows 11

**Table 4:** A comparison of evaliation value in Experiment 2. For each participant and each indicator, we compared the results when the proposed system was used and when it was not, and the results with better results are shown in bold.

Player	System	MAE [cent]	ME [cent]	SD [cent]
A	MIDI Sequencer	24.60	−**20.22**	32.33
	Proposed	**22.98**	−20.25	**26.33**
B	MIDI Sequencer	15.32	**11.05**	31.92
	Proposed	**14.60**	11.45	**23.93**
C	MIDI Sequencer	41.07	−30.30	56.00
	Proposed	**26.99**	−**15.66**	**51.27**
D	MIDI Sequencer	25.13	**9.94**	50.91
	Proposed	**24.98**	14.99	**37.70**
E	MIDI Sequencer	19.53	−**2.77**	38.17
	Proposed	**18.83**	−3.48	**33.81**
F	MIDI Sequencer	20.89	−4.00	47.60
	Proposed	**16.72**	−**3.23**	**35.13**
G	MIDI Sequencer	22.46	**6.00**	**48.19**
	Proposed	**20.71**	7.47	50.60
H	MIDI Sequencer	47.38	−34.51	58.70
	Proposed	**39.08**	−**27.46**	**52.92**
I	MIDI Sequencer	29.01	−13.90	39.77
	Proposed	**22.73**	**1.11**	**39.37**
J	MIDI Sequencer	**36.12**	−**24.86**	50.87
	Proposed	37.18	−25.68	**46.61**
K	MIDI Sequencer	**39.28**	−22.35	**62.24**
	Proposed	40.30	−**16.05**	65.11

## Data Availability

The data are available from the corresponding author on request.

## Supplementary Materials

The following supporting information can be downloaded at: https://www.mdpi.com/article/10.3390/s26031053/s1, Video S1: Video of Performance Using Proposed System.

## References

[1] Kurosawa Y., Suzuki K. Robot-Assisted Playing with Fingering Support for a Saxphone. Proceedings of the International Conference on Mathematics and Computing (ICMC).

[2] Kato T., Shimazaki K., Nundrakwang S., Chuprasert P., Thumwarin P. Proposal of the Concept of a Breathing Assist System for Saxophone Players with Breathing Problems. Proceedings of the 5th International Conference on Engineering, Applied Sciences and Technology (ICEAST).

[3] Kuroda J., Koutaki G. (2022). Sensing Control Parameters of Flute from Microphone Sound Based on Machine Learning from Robotic Performer. Sensors.

[4] Koutaki G., Hamanaka M. Automatic Fingering Saxophone Quartet System. Proceedings of the 24th IFIP TC 14 Entertainment Computing (ICEC).

[5] Chin D., Xia G. A Computer-aided Multimodal Music Learning System with Curriculum: A Pilot Study. Proceedings of the International Conference on New Interfaces for Musical Expression.

[6] Frid E. (2019). Accessible Digital Musical Instruments—A Review of Musical Interfaces in Inclusive Music Practice. Multimodal Technol. Interact..

[7] Matthias R., Graham P., Lagrange M. Analysis of Saxophone Performance for Computer-Assisted tutoring. Proceedings of the International Conference on Mathematics and Computing (ICMC).

[8] Eremenko V., Morsi A., Narang J., Serra X. Performance Assessment Technologies for the Support of Musical Instrument Learning. Proceedings of the 12th International Conference on Computer Supported Education (CSME).

[9] Michałkot A., Di Stefano N., Campo A., Leman M. (2024). Enhancing human-human musical interaction through kinesthetic haptic feedback using wearable exoskeletons: Theoretical foundations, validation scenarios, and limitations. Front. Psychol..

[10] Molero D., Schez-Sobrino S., Vallejo D., Glez-Morcillo C., Albusac J. (2021). A novel approach to learning music and piano based on mixed reality and gamification. Multimed. Tools Appl..

[11] Fazekas G., Wiggins G., Rafee S. Performer Identification From Symbolic Representation of Music Using Statistical Models. Proceedings of the International Conference on Mathematics and Computing (ICMC).

[12] Gingras B., Asselin P., McAdams S. (2013). Individuality in harpsichord performance: Disentangling performer- and piece-specific influences on interpretive choices. Front. Psychol..

[13] MIDI Manufacturers Association (1996). MIDI 1.0 Detailed Specification. https://midi.org/midi-1-0-core-specifications.

[14] Morise M., Kawahara H., Katayose H. Fast and reliable F0 estimation method based on the period extraction of vocal fold vibration of singing voice and speech. Proceedings of the AES 35th International Conference.

[15] Morise M., Yokomori F., Ozawa K. (2016). WORLD: A vocoder-based high-quality speech synthesis system for real-time applications. IEICE Trans. Inf. Syst..

[16] Flanagan J.L., Golden R.M. (1966). Phase Vocoder. Bell Syst. Tech. J..

[17] Morise M. Harvest: A High-Performance Fundamental Frequency Estimator from Speech Signals. Proceedings of the 18th Annual Conference of the International Speech Communication Association, Interspeech.

[18] ITU-R (2015). BS.1770-4: Algorithms to Measure Audio Programme Loudness and Truepeak Audio Level: 2015.

[19] EBU (2014). R-128-Loudness Normalisation and Permitted Maximum Level of Audio Signals.

[20] Alexander L., Claire A., Ashis P., Siddharth G. (2020). An Interdisciplinary Review of Music Performance Analysis. Trans. Int. Soc. Music Inf. Retr..

[21] Chris D., Juan P B., Mike D., Mark S. Complex domain onset detection for musical signals. Proceedings of the 6th International Conference on Digital Audio Effects (DAFx-03).

[22] USB Implementers Forum (2020). Universal Serial Bus Device Class Definition for MIDI Devices, Version 2.0. https://midi.org/usb-midi-2-0.

[23] Okushi K. (2019). Onkyoutyoukakushinnrigaku (Auditory Psychoacoustics).

[24] Adhika S.R., Nur U.M. (2017). Markov Chain Based Procedural Music Generator with User Chosen Mood Compatibility. Int. J. Asia Digit. Art Des..

